# Immersive Innovations: Exploring the Diverse Applications of Virtual Reality (VR) in Healthcare

**DOI:** 10.7759/cureus.56137

**Published:** 2024-03-14

**Authors:** Chaitanya Kumar Javvaji, Harshitha Reddy, Jayant D Vagha, Amar Taksande, Anirudh Kommareddy, Naramreddy Sudheesh Reddy

**Affiliations:** 1 Pediatrics, Jawaharlal Nehru Medical College, Datta Meghe Institute of Higher Education and Research, Wardha, IND; 2 Internal Medicine, Jawaharlal Nehru Medical College, Datta Meghe Institute of Higher Education and Research, Wardha, IND

**Keywords:** surgical skills-based training, advance treatment, cardiology research, advanced medicine, supportive and palliative care, emerging technologies

## Abstract

Virtual reality (VR) has experienced a remarkable evolution over recent decades, evolving from its initial applications in specific military domains to becoming a ubiquitous and easily accessible technology. This thorough review delves into the intricate domain of VR within healthcare, seeking to offer a comprehensive understanding of its historical evolution, theoretical foundations, and current adoption status. The examination explores the advantages of VR in enhancing the educational experience for medical students, with a particular focus on skill acquisition and retention. Within this exploration, the review dissects the applications of VR across diverse medical disciplines, highlighting its role in surgical training and anatomy/physiology education. While navigating the expansive landscape of VR, the review addresses challenges related to technology and pedagogy, providing insights into overcoming technical hurdles and seamlessly integrating VR into healthcare practices. Additionally, the review looks ahead to future directions and emerging trends, examining the potential impact of technological advancements and innovative applications in healthcare. This review illuminates the transformative potential of VR as a tool poised to revolutionize healthcare practices.

## Introduction and background

Virtual reality (VR) is a 3D simulation generated by a computer where individuals engage in interactive experiences. In order to access VR, one often needs a computer that can project 3D data onto different screens or wearable technologies such as head-mounted displays (HMDs). Sensors for user identification improve this even more [1]. As per Sherman and Craig's definition, essential components inherent to the VR experience include a virtual world, interaction, immersion, and sensory feedback [2].

The inception of virtual systems in medicine dates back to 1965 when Robert Mann introduced a platform to facilitate novel training environments for orthopedics [3]. The HMD debuted in the late 1980s [4]. Over 10 years, a novel approach to medical education that prioritizes practical techniques became well-known [3]. In recent years, VR has significantly transformed from limited military applications in the 1970s to widespread accessibility across various sectors. Technological advancements have played a crucial role in this evolution, making VR more affordable and prevalent. Its increased accessibility has led to broader consumer adoption, elevating VR from a niche technology to a widely used and sophisticated tool. This evolution is marked by the capacity to immerse users in virtual environments (VEs) closely resembling reality, attracting attention from consumers and diverse nonconsumer sectors [5].

**Figure 1:**
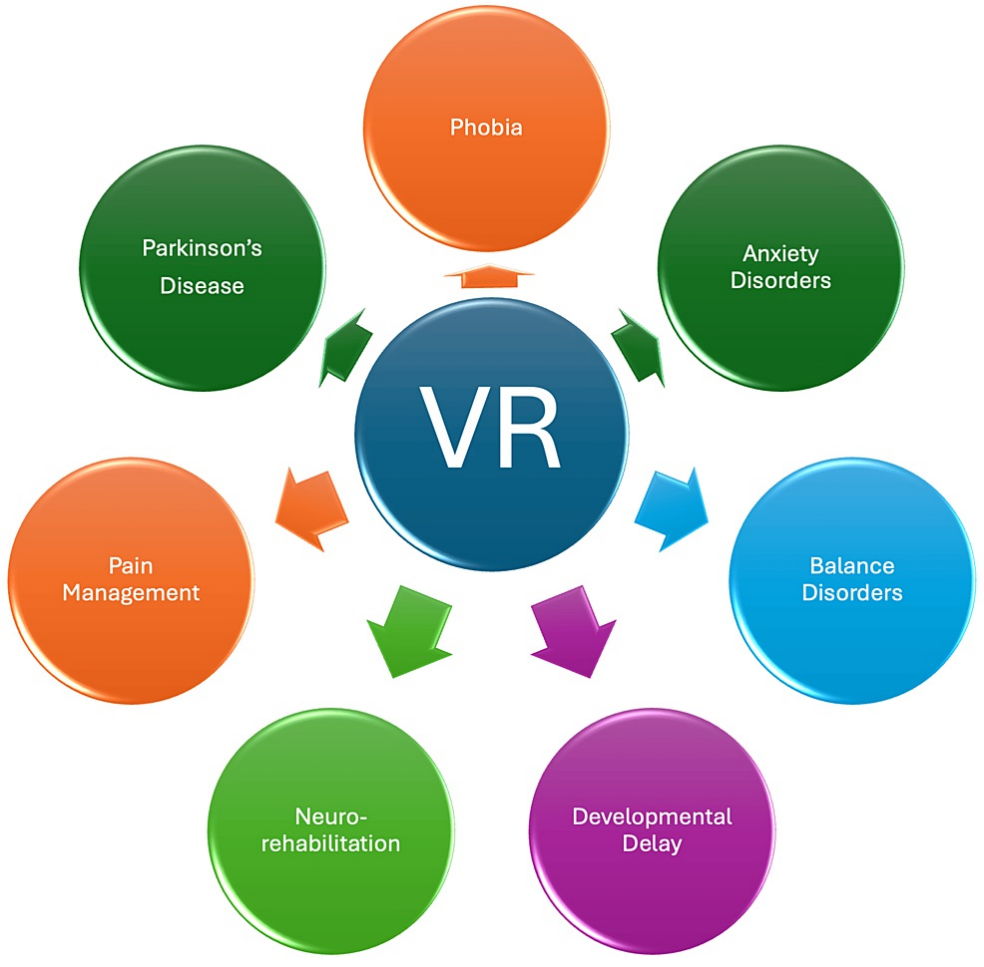
Applications of VR VR: Virtual reality. Source: Chaitanya Kumar Javvaji

A multitude of research studies and controlled trials have presented compelling evidence that underscores the efficacy of VR in attaining therapeutic objectives, especially in phobia and anxiety disorders [6]. VR has demonstrated its efficacy in neurorehabilitation and telerehabilitation for poststroke recovery and balance disorders [7-10]. Moreover, it has shown positive outcomes in addressing Parkinson's disease, pain management, and developmental delay (Figure 1) [11-13]. The robust evidence derived from these investigations underscores the therapeutic potential of VR applications within the medical field. Consequently, VR products emerge as promising tools for medical care, offering the potential to establish markets with favorable returns on investment.

This review endeavors to comprehensively investigate and evaluate the transformative influence of VR in healthcare. The objective of delving into the historical progression of VR integration is to furnish a nuanced comprehension of pivotal milestones and technological advancements. Furthermore, a critical analysis is undertaken to assess the alignment of VR integration with educational theories, evaluate the efficacy of VR applications in clinical training, and propose innovative solutions to address existing challenges. The review seeks to provide insights into the present state of VR adoption in global medical schools, scrutinizing successful implementations and prevalent trends. Additionally, an effort is made to anticipate future directions and emerging trends in VR technology, highlighting its potential to overcome barriers in healthcare practices. Ultimately, this review aspires to contribute valuable knowledge to educators, healthcare professionals, and researchers, fostering a profound understanding of VR's opportunities, challenges, and transformative capacities in these pivotal domains.

## Review

A concise history of VR

The public's and investors' interest in VR and augmented reality (AR) has increased significantly over the last five years, as evidenced by Mark Zuckerberg's $2 billion acquisition of Oculus [14]. Significant investments in VR and AR are being made by prominent companies like Sony, Samsung, HTC, and Google [14]. AR has been used more recently than VR, the topic of study for more than 25 years, and has a large body of literature and a vibrant multidisciplinary academic community [15]. VR research started with computer graphics and spread to other academic fields [16].

Presently, VR-supported video games have gained popularity, serving as valuable tools for researchers in neuroscience, psychology, biology, and other fields. VR enables complex experiments in navigation studies, which would otherwise require researchers to conduct fieldwork with limited intervention possibilities. The study of navigation holds pivotal importance in unraveling the mysteries of human memory, especially in dementia research. This field gained considerable recognition with the 2014 Nobel Prize in Physiology or Medicine awarded to John M. O'Keefe, May-Britt Moser, and Edvard I. Moser for their pioneering work on nerve cells associated with spatial cognition. The term "brain GPS" has been widely adopted in literature to describe this mechanism. Edvard I. Moser has used VR in clinical settings to highlight the technology's significance for clinical practice and research [17]. The availability of free VR tools for experimental and computational use further facilitates accessibility across various domains [18-20].

The ongoing technological revolution has heralded the triumph of 3D environments, expanding the applicability of VR across diverse sectors, including healthcare, military, and education. Apart from its cost-effectiveness, the immersive capabilities of VR contribute significantly to its widespread adoption, serving as a transformative force in enhancing experiences and simulations across various professional domains. As VR progresses, its integration into critical sectors underscores its potential for revolutionizing practices, education, and training methodologies.

In medicine, VR's popularity is rising, with researchers highlighting its potential as an effective tool offering innovative techniques for clinical practice settings. Notably, Morel et al. emphasize the benefits of the VR system in clinical assessment and rehabilitation, pointing to standardization, reproducibility, and stimuli control as key advantages. VR technology provides a standardized virtual environment (VE) where the stimuli can be controlled, facilitating accurate evaluations of patients' balance recovery and progression. This standardized environment enables reproducibility, allowing comparisons among patients in similar conditions or between different patient trials [21].

A comprehensive systematic review by Ravi et al. aligns with this perspective, suggesting that incorporating VR technologies in therapeutic interventions for children and adolescents with cerebral palsy holds promise for improving balance and overall motor capabilities [22]. Additionally, VR technology finds application in psychotherapy, particularly in the treatment of phobias. VR applications have proven effective by exposing patients to controlled, fear-provoking stimuli in a realistic environment, facilitating habituation and extinction processes [23].

Versatility of VR applications in various healthcare disciplines

Recent advancements in teaching tools have introduced a range of technologies to enhance the effectiveness of instructing human anatomy. These include web-based technologies, applications for mobile devices, 3D VR models, and computer-aided learning [24,25]. Among these tools, photogrammetry stands out as a technique enabling the simulation of 3D objects by deriving coordinates and spatial measurements from photographs [26,27].

Incorporating these tools has resulted in noteworthy enhancements in the instruction of human anatomy [28]. In a research by Alharbi et al., medical students conveyed their perspectives on 3D-VR as an educational tool, emphasizing its superior advantages in comprehending human anatomy compared to conventional methods. This tool improved their understanding and retention of knowledge and promoted interaction between educators and students, offering a lifelike, self-directed learning experience [29].

The neurosurgery research laboratory provides an ideal setting for gaining expertise in the intricate anatomy of microsurgery and mastering advanced surgical techniques essential for managing complex neurosurgical conditions. Trainees can utilize state-of-the-art equipment and instrumentation to simulate surgical procedures within this setting. This enables the refinement of visuospatial orientation and fine motor skills and the improvement of surgical techniques, allowing practitioners to enhance their skills before undertaking procedures in the operating room [30].

In a study led by Gonzalez-Romo et al., the importance of interpersonal collaboration in meaningful educational experiences, particularly for instructing intricate micro-neurosurgical anatomy and novel neurosurgical techniques, was underscored. The study proposed multimodal and integrated computerized teaching methods as a viable alternative to conventional cadaver dissection. The research introduced a metaverse, establishing a virtual neuroanatomy laboratory space as a centralized repository for neurosurgery anatomical resources [31].

Motor imagery (MI), a cognitive process involving the mental rehearsal of body movements without physical execution, is a promising poststroke motor rehabilitation strategy [32,33]. However, MI poses challenges, particularly for stroke patients [34]. Augmented by VR technology, action observation (AO) introduces a visually simulated environment that minimizes distractions, potentially alleviating the difficulty associated with conventional MI tasks. This innovative approach, particularly advantageous for stroke patients facing severe motor impairment and limited rehabilitative options, leverages the immersive capabilities of VR to facilitate MI tasks, contributing to enhanced motor recovery [35].

VR offers surgeons an immersive and interactive environment that complements fundamental surgical visualization. A significant benefit of VR is its ability to offer the surgeon real-time visual cues and feedback during the procedure, enhancing surgical precision. Research has demonstrated that VR simulations can lead to a reduction of up to 53.7% in errors related to pedicle screw placement, highlighting its potential to augment surgical accuracy [36].

Integrating technologies such as VR and AR in surgeries addressing spinal deformities proves beneficial in preoperative planning. These technologies offer 3D visualizations of the patient's spinal alignment, providing precise osteotomy landmarks. The use of AR overlays aids surgeons in visualizing the final alignment before making surgical incisions, ensuring more accurate and reproducible surgical procedures. Research indicates that incorporating VR and AR in these surgeries improves patient outcomes, including reduced complications and revisions [37].

VR technology is pivotal in stimulating the brain through high-intensity, multi-sensory, repetitive, and task-oriented feedback, impacting motor, cognitive, and sensory functions. This immersive experience allows patients to engage in virtual environments, optimizing the effectiveness of rehabilitation training. Specifically in multiple sclerosis (MS) patients, VR-based rehabilitation training activates the mirror neuron system, inducing cortical and subcortical changes in the brain. Consequently, this promotes synaptic reorganization and remyelination in the brain's motor areas [38]. Several studies demonstrate how VR-based rehabilitation training promotes synaptic reorganization and remyelination in the brain's motor areas. Research indicates that VR-based therapy improves MS patients' balance, mobility, and cognitive function [39].

MS can lead to profound functional impairments. To address this, rehabilitation training utilizing VR technology enhances cognitive function, movement, and balance in MS patients. MS patients were split into two groups in research by Munari et al.: one group received VR in addition to robot training, while the other group just received robot training. After six weeks of rehabilitation, Berg Balance Scale (BBS) scores were considerably higher in the VR plus robot training group than in the robot training alone group [40]. On the other hand, Casuso-Holgado et al. reviewed five studies that used the BBS as an evaluation instrument in their analysis of VR-based balance rehabilitation training for patients suffering from MS, and there was no statistically significant variation [41].

To find out how VR training affected the gait of people with MS, Peruzzi et al. carried out a single-blind randomized control experiment. While the experimental group received VR-enhanced treadmill exercise, the control group received standard treadmill training. The results showed that both groups had significantly improved walking endurance, speed, stride length, lower limb joint strength, frequency, and range of motion. Interestingly, the experimental group's improved balancing function was noticeably more than that in the control group [42].

Maggio evaluated the impact of VR training on motor and cognitive disorders in individuals with MS. The assessment utilized neuropsychological and clinical scales, revealing a noteworthy improvement in motor scores and cognitive parameters [43]. Jonsdottir et al. employed VR technology in their study to target motor impairment in the upper limbs of MS patients. The results suggested that VR can potentially improve upper limb motor function in individuals with neurological disorders, presenting valuable opportunities for home-based rehabilitation [44].

VR can potentially be used in teaching and training medical practitioners, especially in intensive care units (ICUs) where complex treatment plans require theoretical understanding and preparedness for practice [45]. In a randomized controlled trial conducted by Nas et al., the efficacy of VR in teaching cardiopulmonary resuscitation was assessed among 381 participants. Although the VR group's chest compression rates were not as high as those of face-to-face training, they were nonetheless lower. Significant variability may be seen in this field's VR/AR research environment [46]. To improve conventional extracorporeal membrane oxygenation (ECMO) training methods, Wolff et al. creatively created a VR training environment [47].

Bronchoscopy, used in patients, is a diagnostic and therapeutic procedure with some learning difficulty. Colt et al. tackled this issue by developing a VR bronchoscopy simulation. After receiving VR training, inexperienced doctors in the model demonstrated dexterity, speed, and accuracy like experienced physicians [48]. Chiang et al. examined the effects of a 15-minute VR-based learning session on tracheostomy care in prospective randomized research including 60 healthcare personnel. After utilizing VR materials, participants experienced significant enhancements in self-efficacy, with favorable effects persisting for three to four weeks. This encompassed increased familiarity, elevated self-confidence, and reduced anxiety levels [49].

Stress is an essential issue in the ICU, affecting patients and medical staff. Nijland et al. investigated the efficacy of VR in reducing perceived stress levels in a study that included 66 ICU nurses. The findings showed that 62% of ICU nurses who used VR relaxation during time off thought it was beneficial for lowering stress [50]. It was also shown by Bodet-Contentin et al. that using VR to increase the efficacy of breaks might benefit 88 carers in the ICUs [51].

Jawed et al. conducted a study involving 15 ICU patients to assess the potential of VR in mitigating deprivation and sensory overload in the ICU environment. Using VR goggles, patients saw calming footage of beaches with natural sound effects for 15 minutes. Most patients said that VR treatment reduced their anxiety and tension and that they could tolerate the headgear well [52]. Naef et al. conducted a study investigating the optimal auditory and visual stimuli duration for patients in the ICU. Research indicates that visual stimulation should not last more than 10-15 minutes to avoid adverse side effects, and audio stimuli should not last longer than an hour [53].

Using VR as an intervention or treatment is considered a part of palliative care (PC), with the primary goal being to provide patients with additional psychosocial support. VR interventions are usually brief and provide leisure or relaxation experiences, emphasizing boosting emotional well-being, improving quality of life, offering a cognitive distraction, and acting as an adjuvant therapy to reduce psychological symptoms like pain, anxiety, and depression [54-57]. According to some studies, VR is considered a "therapeutic intervention," which suggests that it is an evidence-based strategy that therapists or intervention professionals frequently assist. Helmchem highlights that determining an intervention's efficacy requires comparing its results to its intended therapeutic outcome [58].

There is a compelling need for more extensive longitudinal investigations to systematically evaluate the effectiveness of VR-based therapeutic interventions in PC. Given the imperative of delivering comprehensive, individualized attention to address the multifaceted physical and psychological requirements of patients with advanced, life-limiting illnesses, such studies play a crucial role. They establish realistic expectations for intervention outcomes and facilitate the appropriate involvement of healthcare professionals in the patient's care.

Pulmonary rehabilitation is a therapeutic approach centered on breathing exercises to improve lung function and alleviate symptoms associated with chronic lung diseases, including chest tightness, chronic cough, and wheezing. Pulmonary rehabilitation is often utilized not just for lung diseases but also for the treatment of hypertension, chronic pain, and cardiovascular disorders, which include illnesses such as myocardial infarction, arrhythmia, and coronary artery disease [59,60].

Furthermore, breathing techniques have shown promise in offering straightforward yet efficient treatments for anxiety, depressive episodes, and other mental health-related conditions [61,62]. In recent years, VR technology has been widely used in various medical applications. This includes treating mental health issues, including anxiety and stress-related problems, in addition to lung diseases like lung cancer, chronic obstructive pulmonary disease (COPD), and asthma [63,64].

Numerous research studies have created diverse virtual environments (VEs) to enhance respiratory function through specialized breathing exercises [65-67]. Another study centered on a VR intervention targeting dyspnea in COVID-19 pneumonia patients. This intervention utilized a virtual room featuring a gender-matched virtual body synchronized with the patient's chest movements [67].

An innovative approach entailed using multi-user VR, immersing participants in an underwater environment featuring jellyfish and a growing glass sponge to facilitate synchronized breathing and heightened awareness. This scenario used a jellyfish to metaphorically represent each participant's breath, giving clear visual input. As participants' breathing synchronized, the glass sponge grew and emitted light. Another noteworthy project, the Bubble Tower, explored the incorporation of breathing techniques into VR gameplay mechanics. This initiative presented a collection of mini-games designed to train participants in fundamental breathing skills. These games included popping bubbles with a forceful breath and extinguishing candles with a prolonged breath [68].

Moreover, a distinctive VE inspired by the escape room game philosophy was developed, challenging participants to employ various breathing patterns to accomplish tasks and successfully escape the room. The tasks within this VE spanned activities such as extinguishing candles, inflating balloons, and operating a toy gun, with each interaction contributing to the participant's respiratory training. Collectively, these studies underscore a range of innovative approaches in utilizing VEs for respiratory exercises. They demonstrate creativity in design and interactivity, emphasizing the potential to enhance breathing awareness and control through diverse and engaging methodologies [68].

Our vast library of more than 400 clinical VR Models (VRMs) at the Children's Hospital of Illinois was created especially for preoperative planning. Initially applied in congenital cardiac surgery, this immersive experience has progressively extended to encompass various other surgical disciplines. When the minute intricacies of 3D anatomy and orientation are crucial considerations in medical decision-making, treating doctors can benefit most from 3D modeling. Congenital cardiac conditions, with their diverse and unique orientations, offer an optimal substrate for the impactful application of VRM. Other medical centers have reported similar experiences and benefits, particularly in cardiothoracic surgery, where proceduralists express an increasing demand for such technology over time [69,70].

The initial foray into cardiac applications has significantly shaped our understanding of the impact of VRM, guiding subsequent efforts. Moreover, neurosurgeons use 3D representations in a variety of ways. Therefore, neurosurgical applications offer a significant possibility for VRM. In cases of brain tumors, VRM aids in precise localization with a high degree of accuracy. Stereo electroencephalography (SEEG) has become more widely used for anatomical mapping in epileptic patients, and VR modeling has been essential in improving presurgical planning and patient education, especially in detecting centers of activity [71].

It might be difficult for patients and parents to grasp medical operations since surgeons' 3D visualization of complicated anatomy varies. The creation of 3D models using VR technology has emerged as a valuable tool to significantly enhance communication, facilitating shared decision-making in the medical context [72]. We are presently gathering survey data across our two distinct centers to assess the experiences of patients and parents when VR models are incorporated into the preoperative education and informed consent processes. Positive responses and remarks have been received right away.

VR benefits patient communication, clinician training, and simulation. It allows healthcare professionals to acquire and develop experience outside direct patient care. This is especially important in high-risk medical settings like trauma, emergency, or surgical care. Given restrictions on resident duty hours, VR simulations offer a realistic and effective alternative to traditional methods like role-playing. In scenarios like the cardiac ICU, VR simulation has proven substantially more realistic, replicating clinical situations effectively [73].

Various simulators have been developed for neurosurgical and pediatric surgery training [74-76]. Patients also benefit from VR; for example, individuals with hand burns experienced significant improvements in the functioning of the hand, performance of an activity, and satisfaction when utilizing VR compared to those undergoing traditional rehabilitation [77]. Similarly, children with cerebral palsy exhibited notable motor function and balance improvements through a VR-incorporated horse-riding simulator [78].

Real-time intraoperative computer vision analysis holds significant promise for enhancing surgical safety, offering AR support to surgeons during procedures. Published computer vision models have demonstrated their capacity to evaluate operative complexity, aid in decision-making for minimally invasive procedures, objectively assess surgeons' technical skills, provide real-time feedback during surgery, evaluate dynamics within the operating room team, and even predict postoperative outcomes based on intraoperative events [79].

James et al. demonstrated the superior accuracy and intuitiveness of VR-assisted transseptal puncture compared to fluoroscopy-guided procedures, particularly for less-experienced operators [80]. The development of VR-based skills simulators extends to various medical procedures, including angiography, vascular access, percutaneous valve implantation, surgeries, and electrophysiology [81-83]. VR is a priceless instrument for providing novice operators comprehensive training before encountering real-world scenarios, including the endovascular procedure for abdominal aortic aneurysms. VR serves as a priceless instrument [84]. Successful VR-based resuscitation training implementations have also been documented [85].

The onset of the COVID-19 pandemic disrupted traditional medical practices, creating challenges for in-person visits. VR emerged as a pivotal solution, offering immersive rehabilitation experiences with minimal exposure risk. As telemedicine becomes more prevalent, allowing remote sharing of patient data and consultations with healthcare professionals, VR facilitates virtual consultations with experts, overcoming time and space constraints. Research indicates that VR-based remote multidisciplinary meetings are feasible and engaging, highlighting telemedicine's enduring significance beyond the pandemic [86].

Limitations

Despite the increasing number of studies on VR applications in healthcare, systematic synthesis efforts, such as the present study, face challenges due to the limited comparability of methods, devices, and protocols [87]. Several potential biases impact the validity of studies investigating VR and AR in the ICU. Selection bias is caused by small, nonrepresentative sample sizes. People in the geriatric age group are constrained by their unfamiliarity and potential resistance to these technologies and, hence, are excluded from the studies. VR interfaces must accommodate age-related changes, including hearing and visual impairment adjustments, to ensure broad accessibility and usability.

High-performance bias is expected due to the lack of blinding in studies, influencing participant behavior. Subjective outcomes and dependence on observations further add complexity, necessitating the establishment of objective evaluation criteria. However, certain conditions, like posttraumatic disorders, present inherent complexity, making it challenging to develop comprehensive evaluation criteria. The diversity of VR systems poses a common challenge, affecting application complexity and user tolerance. Variations in performance, user comfort, and immersion levels make data reproducibility difficult, impacting research reliability and comparability [88].

Integrating VR into daily medical practice faces several challenges. The foremost obstacle is the high cost of VR software, logistics, hardware, and personnel, making VR projects financially inaccessible for many institutions. However, emerging affordable options, such as the Google headset and free, open-source software like Slicer 3D, promise to overcome economic barriers, especially in resource-limited environments. Another significant challenge is the limited evidence supporting the efficacy of VR in medical settings, with most studies conducted in pioneering centers needing more methodological standardization and robust evidence from multicenter trials. The lack of FDA-approved medical devices hinders widespread acceptance and funding for VR projects.

Additionally, the development of VR technologies has been primarily driven by the entertainment industry, needing more input from physicians and patients. Reliability issues, including durability in extreme medical environments and user-friendly interfaces for interventionists and patients, pose critical hurdles. As VR technologies progress, future studies must demonstrate their clinical benefits and establish their reliability in everyday healthcare practices.

Future prospects of VR in healthcare

VR holds great promise in the field of rehabilitation and therapy. Tailored VR experiences can assist in physical therapy, mental health interventions, and rehabilitation exercises. VR's interactive and engaging nature can motivate patients to adhere to their treatment plans, improving outcomes. VR has the potential to revolutionize telemedicine by offering a more immersive and interactive experience for remote consultations. Patients can use VR to share real-time health data, engage in virtual consultations with healthcare professionals, and participate in remote monitoring programs, increasing accessibility to quality healthcare. VR can enhance preoperative planning and intraoperative navigation. Surgeons can utilize VR to visualize patient-specific anatomy in three dimensions, practice complex procedures, and navigate through intricate anatomical structures during surgery. This can lead to improved surgical precision and patient outcomes. VR facilitates collaborative decision-making among healthcare professionals. Multidisciplinary teams can use VR to review and discuss complex cases, share insights, and plan interventions in a virtual space. This can improve communication and coordination, particularly in cases involving diverse specialists.

## Conclusions

VR presents a transformative influence on healthcare, with applications ranging from medical education to surgical training, therapeutic interventions, and remote patient care. The immersive nature of VR enhances learning experiences and contributes to improved patient outcomes. Overcoming challenges such as accessibility for diverse populations is crucial for widespread adoption. Ongoing research and collaboration promise a future where VR becomes integral to personalized, effective, and accessible healthcare. The potential impact of VR in healthcare is profound, offering innovative solutions and elevating the overall quality of patient care.
